# FAK auto-phosphorylation site tyrosine 397 is required for development but dispensable for normal skin homeostasis

**DOI:** 10.1371/journal.pone.0200558

**Published:** 2018-07-12

**Authors:** Joel B. Heim, Cera A. McDonald, Saranya P. Wyles, Sindhuja Sominidi-Damodaran, Edwin J. Squirewell, Ming Li, Catherine Motsonelidze, Ralph T. Böttcher, Jan van Deursen, Alexander Meves

**Affiliations:** 1 Department of Dermatology, Mayo Clinic, Rochester, Minnesota, United States of America; 2 Department of Molecular Medicine, Max Planck Institute for Biochemistry, Martinsried, Germany; 3 German Center for Cardiovascular Research-Munich Partner Site, Munich, Germany; 4 Department of Biochemistry and Molecular Biology, Mayo Clinic, Rochester, Minnesota, United States of America; 5 Department of Pediatric and Adolescent Medicine, Mayo Clinic, Rochester, Minnesota, United States of America; 6 Mayo Clinic Cancer Center, Mayo Clinic, Rochester, Minnesota, United States of America; Technische Universitat Dresden, GERMANY

## Abstract

Focal adhesion kinase (FAK) is an intensely studied non-receptor tyrosine kinase with roles in cancer and other common human diseases. Despite the large interest in FAK, the in vivo contribution of FAK auto-phosphorylation site tyrosine (Y) 397 to FAK function is incompletely understood. To study FAK Y397 in vivo we analyzed mice with ‘non-phosphorylatable’ Y-to-phenylalanine (F) and ‘phospho-mimicking’ Y-to-glutamate (E) mutations in the germline. We found that FAK Y397F mice die early during embryogenesis with abnormal angiogenesis like FAK kinase-dead mice. When Y397 is mutated to a glutamate mice survive beyond mid-gestation like mice where Y397 is lost by deletion of FAK exon 15. In culture, defects in proliferation, invasion and gene expression were more severe with the FAK Y397F than with the FAK Y397E mutation despite the inability of FAK Y397E to bind SRC. Conditional expression of FAK Y397F or Y397E in unchallenged avascular epidermis, however, resulted in no appreciable phenotype. We conclude that FAK Y397 is required for the highly dynamic tissue remodeling during development but dispensable for normal homeostasis of avascular epidermis. In contrast to the Y397F mutation, FAK Y397E retains sufficient biological activity to allow for development beyond mid-gestation.

## Introduction

Focal adhesion kinase (FAK) is a non-receptor tyrosine kinase [1] enriched in focal adhesions [2]. FAK has been shown to play important roles in diverse events such as cell adhesion[3], wound healing [4], and metastasis [5]. FAK has also been localized to the nucleus, where it may influence cell survival [6] and modulate gene transcription [7].

The structure of FAK consists of a focal adhesion targeting (FAT) domain on the C-terminus, an adjacent catalytic kinase domain and a four-point one, ezrin, radixin, moesin (FERM) domain on the N-terminus. The central catalytic and FERM domains are connected by a linker region which contains the major FAK auto-phosphorylation site, tyrosine (Y) 397 [1]. When FAK is in the inactive conformation, the FERM domain auto-inhibits the catalytic kinase domain and Y397 is nonphosphorylated [8]. Upon activation, the linker region becomes exposed and Y397 undergoes autophosphorylation [8], providing a high-affinity binding site [9] for proteins such as proto-oncogene tyrosine-protein kinase Src (SRC) [10].

Although much is known about the mechanisms and biological activities of FAK in vitro, the regulation of FAK in vivo is incompletely understood. FAK-null mice experience embryonic lethality by embryonic day (E) 8.5 [11, 12]. Deletion of FAK Y397 by linker domain truncation mutation results in embryonic death between E13.5-E14.5 [13], while the substitution of Y397 with a non-phosphorylatable phenylalanine (Y397F) results in embryonic death by E11.5 [10]. Conditional FAK deletion has been shown to suppress tumor formation in breast epithelium [14] and the epidermis [15]. Further, phosphorylated Y397 FAK has been localized in the nuclei of cancerous cells, including melanoma [10] and colorectal cancer [16]. These findings suggest that FAK Y397 plays an important role during embryonic development and may influence cell survival and gene transcription in cancerous cells, however may not be necessary for cell survival in mature differentiated cells.

Here, we explored the role of FAK Y397 in embryonic development and mature differentiated epidermis by comparing mice with Y397-to-phenylalanine (Y397F) and Y397-to-glutamate (Y397E) germline mutations, allowing us to examine the in vivo activities of FAK lacking a phosphorylatable tyrosine (Y397F) or harboring a constitutive negative charge at Y397 (Y397E).

## Materials and methods

### Mouse strains

Targeting of embryonic stem cells and mouse chimera production was through the Mayo Clinic Transgenic Core Facility (Rochester, MN). FAK Y397F mice have been reported previously [10]. FAK^fl/fl^ mice [17] were obtained from the Mutant Mouse Regional Resource Center at UC Davis. Keratin 5-Cre mice were as previously described [18]. To humanely and efficiently euthanize animals carbon dioxide (CO2) was used following American Veterinary Medical Association guidelines. All animal experiments were approved by the Institutional Animal Care and Use Committee at Mayo Clinic, Rochester, MN, and all experiments were performed in accordance with relevant guidelines and regulations.

### Antibodies

The following antibodies were used for immunoprecipitation and/or microfluidic western blot analysis by ProteinSimple (PS), immunohistochemistry (IHC), immunofluorescence (IF): CD31 (553371, MEC13.3, BD Biosciences; 1:500 for IF), laminin (ab11575, Abcam; 1:400 for IF), involucrin (ab28057, Abcam; 1:100 for IHC), FAK (06–543, EMD Millipore; 1:50 for PS; 1:100 for IF), paxillin (610051, 349, BD Biosciences, 1:100 for IF), phospho-FAK Y397 (AF4528, R&D Systems; 1:200 for PS), phospho-FAK Y397 (44-624G, Thermo Fisher Scientific; 1:100 for IF), β-tubulin (ab15568; Abcam; 1:50 for PS), SRC (2109, 36D10, Cell Signaling Technology; 1:50 for PS), phospho-SRC Y416 (2101, Cell Signaling Technology; 1:10 for PS).

### Plasmids and constructs

For stable FAK variant expression in cell lines, cDNA was cloned into the lentiviral expression vector LV022 (Applied Biological Materials). Point mutations were introduced by site-directed mutagenesis using the Quikchange II XL kit (200521, Agilent Technologies).

### Primary cells and cell lines

Heterozygous FAK Y397F mice were intercrossed and MEFs were isolated from E9.5 embryos, immortalized with the SV40 large T antigen. FAK Y397F mutation was confirmed by RNA sequencing. Primary mouse keratinocytes were isolated and cultured as previously described^30^. p53-/- FAK knock-out mouse embryonic fibroblasts (MEFs; CRL-2644) were purchased from the American Type Culture Collection. Cell lines were tested for mycoplasma and viral contamination by the RapidMAP^™^ 21 test (Taconic) or the Mayo IMPACT Profile (IDEXX BioResearch). MEFs were cultured in DMEM containing 10% FBS.

### Transient and stable transfection/transduction

To generate stable cell lines, MEFs were infected with lentivirus in 2 ml complete medium containing 8 μg/ml polybrene and a virus multiplicity of infection (MOI) of 2 to 20. Selection was by 1 to 1.5 μg per ml of puromycin.

### Timed mating and embryo isolation

In all mating procedures, female mice were exposed to male mice overnight. Identification of a vaginal plug the next morning was used to determine embryonic day (E) 0.5. Staged embryos (E8.5-E16.5) were dissected in ice-cold PBS. Embryos were observed under an Olympus SZX12 Microscope. Small pieces of tail or paw were digested in PBS containing proteinase K at 56 °C for 90 min. Proteinase K was heat-inactivated at 80 °C for 30 min. 2 μl of the sample were used to set up a PCR reaction for genotyping.

### Whole-mount three-dimensional imaging of CD31 in embryos

Whole-mount three-dimensional imaging of CD31 was performed as previously described[18]. To allow for deep penetration of laser light and confocal sectioning, embryos were dehydrated by increasing methanol concentrations, cleared in benzyl alcohol/benzyl benzoate (1:2). Images were obtained with a Zeiss LSM780 confocal microscope using ZEN software (2012, release 8.0).

### Peptide pulldowns

Pulldowns were performed as previously described [19]. Purified GST-tagged SRC was purchased from Sigma-Aldrich (S1076).

### Microfluidic western blotting

Western blots were performed as Simple Western^™^ assays using the Wes system (ProteinSimple), a combination of capillary electrophoresis and immunodetection techniques, following the manufacturer’s protocols. Quantification of chemiluminescence was based on peak height after correction for a baseline signal. Raw data was generated by the Compass software (version 2.5.8 to 2.7.1, build ID 0201–0826). Compass is the control and data analysis application for Simple Western instruments.

### Proliferation and Matrigel invasion

For proliferation analysis, cells were seeded at low densities (2,500 to 5,000 cells per well). Cells were incubated using an IncuCyte ZOOM^®^ (Essen Bioscience). Cell growth based on percent confluence was determined from phase contrast images. For proliferation assays based on automated counting of fluorescent nuclei, cells were first infected with the NucLight Red reagent (4476, Essen Bioscience) and selected using 1 μg per ml of puromycin to obtain a stable nuclear red fluorescent label. Invasion assays were performed by seeding cells at a density of 30,000 cells/well on a thin coating of Matrigel (0.1 mg/ml, standard formulation, Corning) in 96 well ImageLock microplates (4379, Essen BioScience). Cells were then allowed to grow confluent. Prior to scratching, cell proliferation was inhibited by 30 minutes exposure to 10 μg/ml mitomycin C (M4287, Sigma-Aldrich). Monolayers were scratched using the WoundMaker^™^ pin tool (4493, Essen BioScience). Medium was changed and cells were covered in 2 mg/ml Matrigel (standard formulation, Corning) diluted in complete medium. Relative wound density was calculated using the IncuCyte^™^ Scratch Wound Cell Migration Software Module (9600–0012).

### Adhesion turnover

LentiBrite^™^ Paxillin (Millipore)-GFP-tagged FAK Y397E and FAK Y397F cells were plated (10,000 per well) in regular growth medium in Lab-Tek II chambered coverglass (Nunc) coated with 10 μg/ml of fibronectin (Calbiochem). The focal adhesion turnover analysis was performed on a custom-made system (Visitron Systems) based on an Nikon Eclipse Ti inverted microscope, an Nikon 60x 1.40 NA objective and a ProEM EMCCD camera (Princeton Instruments) in an incubation chamber (Oxolab) to control temperature, CO2 (5% premixed) and humidity. Images were acquired every three minutes for four hours using VisiView software (Visitron Systems) and analyzed by ImageJ.

### Immunocytochemistry and immunohistochemistry

For immunocytochemistry, cells were cultured on LAB-TEK II chamber slides (154453, Thermo Scientific), fixed for 10 min in 4% PFA at room temperature and stained with a BOND-MAX autostainer (Leica Biosystems). Briefly, slides were washed with 1x Bond Wash solution (Leica Biosystems) and cells were permeabilized for 10 min with 0.1% Triton X-100 in PBS. After washing and blocking with 3% BSA in PBS for 60 min, slides were incubated with a 1:100 dilution of primary antibody in Bond Primary Antibody Diluent (Leica Biosystems) for 90 min. Slides were washed three times for 2 min and incubated with a 1:100 dilution of secondary antibody in BOND Primary Antibody Diluent for 60 min. After three 2 min wash steps, DAPI staining was performed for 10 min. Images were collected at room temperature by confocal microscopy (Zeiss LSM780) with a x40/1.0 objective using ZEN software (2012, release 8.0). For immunohistochemistry on paraffin sections using involucrin antibody, sections were baked, dewaxed and exposed to EDTA based pH 9.0 solution (AR9640, Leica Biosystems) using a BOND-MAX autostainer. Antibody detection was by alkaline phosphatase-linked polymers (DS9390, Leica Biosystems). For immunohistochemistry on frozen murine skin sections, epitope retrieval was not required. Antibody detection was by secondary Alexa 488-conjugated antibody (1:200 dilution). Hematoxylin and eosin-stained paraffin sections were digitalized with an Aperio ScanScope (Leica Biosystems).

### Experiments on micropatterns

FAK variant cells were seeded on fibronectin-coated micropatterned discs (10-900-10, CYTOO) according to instructions and allowed to attach and spread for 1 hour. Cells were then fixed for 10 min in 4% PFA at room temperature and processed for immunocytochemistry. Images were collected at room temperature by confocal microscopy (Zeiss LSM780) with a x40/1.0 objective using ZEN software (2012, release 8.0). Reference cells were created using a CYTOO-provided image analysis macro (CYTOOL-IP-Reference cell/April 2014) in ImageJ (release 1.44i).

### Gene expression by next-generation sequencing

RNA was isolated using the RNeasy Plus Mini kit (74134, Qiagen). RNA sequencing was performed as previously described [20]. Briefly, RNA-derived cDNA libraries were prepared using the TruSeq RNA Library Prep Kit v2 (Illumina). Concentration and size distribution of the resulting libraries were determined on an Agilent Bioanalyzer DNA 1000 chip and confirmed by Qubit fluorometry (Life Technologies). Unique indexes were incorporated at the adaptor ligation phase for three-plex sample loading. Libraries were loaded onto paired end flow cells to generate cluster densities of 700,000 per mm^2^ following Illumina’s standard protocol. The flow cells were sequenced as 51 paired end reads on an Illumina HiSeq 2000. The samples were processed through the Mayo RNA-Seq analysis pipeline, MAP-RSeq. Raw and normalized (read per kilobase of gene per million mapped reads) gene expression read counts were obtained per sample. Differential gene expression analysis was carried out using the freely available edgeR bioconductor software package (http://bioconductor.org). Because scaling by total lane counts can bias estimates of differential expression, edgeR uses trimmed mean normalization on raw read counts to determine whether genes are differentially expressed using the negative binomial method. The Benjamini and Hochberg correction is used to control for multiple testing to obtain a false discovery rate.

### Statistics

Statistical analysis was performed using the GraphPad Prism software (version 6.05, GraphPad Software). Statistical significance was determined as indicated.

## Results

### Successful generation of FAK Y397 mutant mice

**Fig 1:**
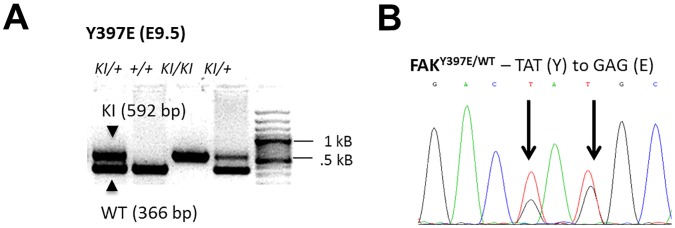
Generation of FAK Y397 mutant FAK. (A) Generation of hetero- and homozygous mutants was confirmed by PCR of E9.5 embryos. Arrows indicate bands representing mutant (Kl) or WT alleles. +, wild-type; Kl/+, heterozygous mutant; Kl/Kl, homozygous mutant; bps, base pairs. (B) Sanger sequencing was performed with mouse tail cDNA to detect heterozygous FAK^Y397E/WT^ mutants. Arrows indicate sites of single base pair mutagenesis.

To study the phenotype of mice expressing FAK Y397F or Y397E during development and in adult tissue, we targeted exon 15 of the murine FAK gene by homologous recombination in embryonic stem (ES) cells. Positive ES cell clones of each mutation were injected into blastocysts to establish the mutant knock-in (KI) mouse strains (KI^neo+^) [10]. The Flippase Recombination Target (FRT)-flanked neomycin gene was then removed by intercrossing the mutant mouse strains with a deleter-Flippase strain, resulting in mice carrying an intronic FRT site and defined point mutations in exon 15 of the FAK gene (KI^frt^). Heterozygous intercrosses of FAK Y397F [10] and Y397E mice (Table 1) yielded no live homozygous offspring at birth. However, homozygous embryos could be detected by PCR genotyping on embryonic (E) day 9.5 (Fig 1A). Sanger sequencing of cDNA derived from the tails of adult heterozygous KI^frt^ mice showed double peaks at amino acid codon 397, indicating base pair changes introduced by homologous recombination (Fig 1B).

**Table 1:** Genotypes of embryos obtained from crosses between FAK Y397E/+ heterozygous mice derived from ES cell clones 227 and 405.

Genotype	Clone 227						Clone 405
	9.5	10.5	13.5	14.5	16.5	Born (P0)	Born (P0)
+/+	5	10	12	15	12	211	13
+/Y397E	11	8	19	38	25	432	26
Y397E/Y397E	5	18	10	15	0	0	0
No. of litters	2	4	5	7	4	100	8

The total number of embryos of each genotype obtained from crosses between mice (FAK Y397E/+; ES cell clones 227 and 405) is shown at various embryonic days (E) and at birth (P0).

### FAK Y397F mice die during early mesoderm development whereas FAK Y397E mice display abnormalities after mid-gestation

**Fig 2:**
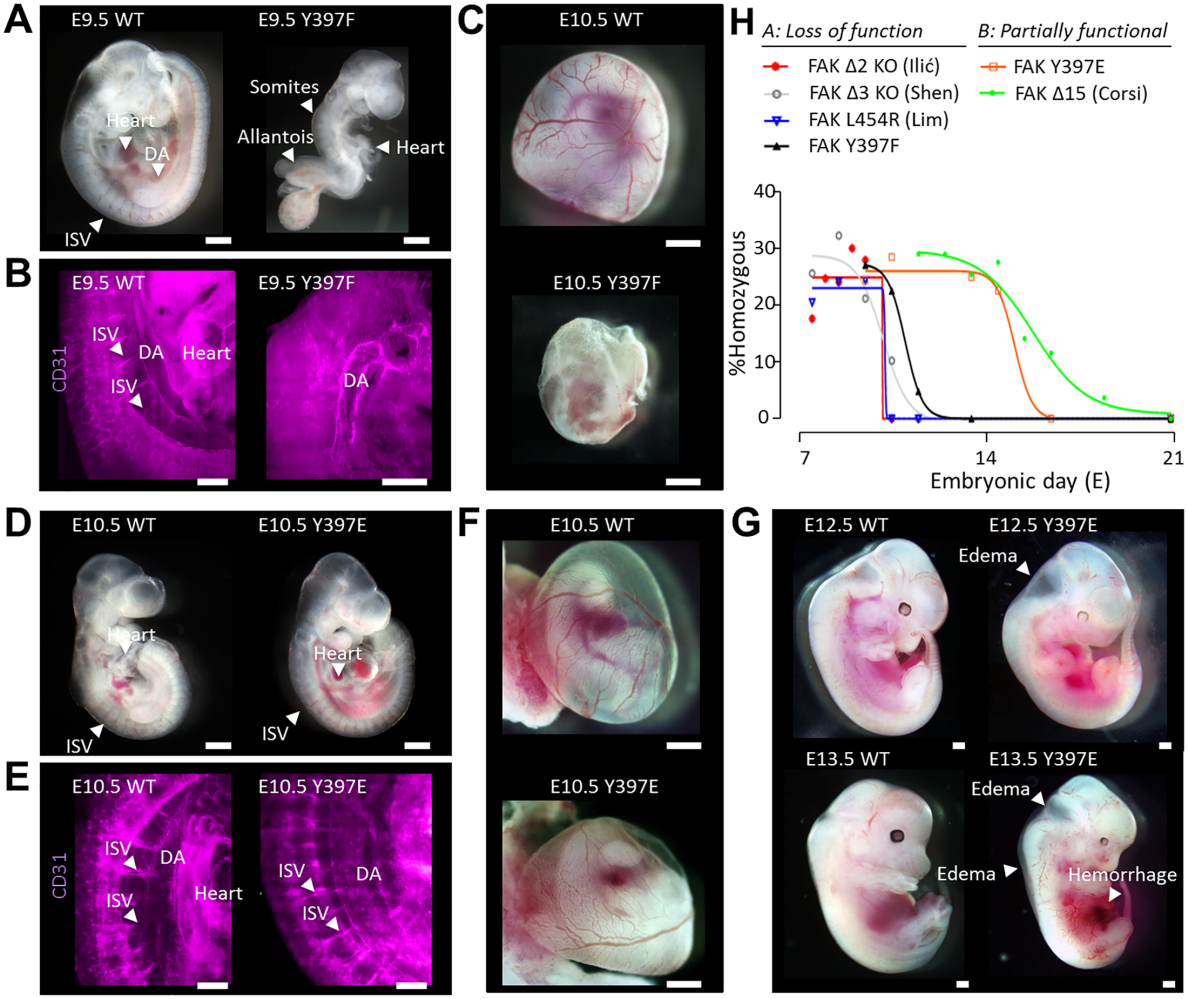
Y397E FAK embryos survive longer than Y397F FAK embryos. (A) Bright-field images of E9.5 WT and Y397F FAK embryos. Arrows point to heart, ISVs, DA, somites, and allantois. WT, wild-type; ISV, intersomitic vessels; DA, dorsal aorta. (B) Whole-mount 3D imaging of CD31-stained E9.5 WT and Y397F FAK embryos. Arrows point to DA, ISVs, and heart. 3D, three-dimensional; CD31, platelet endothelial cell adhesion molecule (PECAM-1). (C) Bright-field images of E10.5 WT and Y397F FAK yolk sacs. (D) Bright-field images of E10.5 WT and Y397E FAK embryos. Arrows point to ISV and heart. (E) Whole-mount 3D imaging of CD31-stained E10.5 WT and Y397E FAK embryos. Arrows point to ISV, DA, and heart. (F) Bright-field images of E10.5 WT and Y397E FAK yolk sacs. (G) Bright-field images of E12.5-E13.5 WT and Y397E FAK embryos. Arrows point to edema and hemorrhage. (H) Survival rate of homozygous FAK mutants by embryonic day, with class of mutant and specific mutation indicated. Scale bars, 200 μm.

Timed mating revealed that FAK Y397F mice die between E9.5 and E11.5 [10] whereas FAK Y397E mice survive until E14.5 (Table 1). No live embryos were seen at E16.5. FAK Y397F embryonic development stalled at E9.5, a stage at which intersomitic vessels (ISVs) were clearly visible between the somites of wild type (WT) but not FAK Y397F embryos (Fig 2A). Mutant embryos demonstrated malformed hearts and an unorganized somite structure while the allantois was unfused and enlarged [10]. Whole-mount three-dimensional (3D) imaging of platelet endothelial cell adhesion molecule-stained (PECAM-1; CD31) Y397F FAK embryos indicated the presence of a dorsal aorta (DA) adjacent to an incompletely formed heart, and a lack or malformation of ISVs, as compared to the clearly stained and normally developed heart, DA, and ISVs of the WT embryo (Fig 2B). Additionally, the FAK Y397F yolk sacs lacked the differentiated blood vessels seen on WT yolk sacs (Fig 2C).

FAK Y397E embryos in contrast were comparable in size and organ development to wild type (WT) embryos at E10.5 with visible and well-defined ISVs (Fig 2D). Whole-mount 3D imaging of PECAM-1-stained FAK Y397E embryos demonstrated developing vascularization as well as an identifiable DA and ISVs (Fig 2E). The yolk sac showed differentiated blood vessels (Fig 2F). At E12.5, however, embryos demonstrated edema, and by E13.5 both edema and hemorrhage were clearly visible (Fig 2G). Compared to published FAK mutant mice [11–13, 21] the in utero survival curve of FAK Y397E embryos resembled that of mice with a 19 amino acid FAK linker deletion (FAK Δ15) (Fig 2H) [13]. In contrast, the FAK Y397F mutation was early embryonic lethal akin to FAK-null or kinase-dead mutations (Fig 2H) with a phenotype resembling fibronectin (FN)-deficient embryos [22]. Together, our data suggested that Y397F sustained a non-active FAK conformation in vivo, while Y397E was partially functional with a phenotype similar to FAK Δ15 mice. FAK Y397 mutants may be broadly classed into loss of function mutations, including Y397F FAK, and those that are partially functional, including Y397E FAK and FAK Δ15 (Fig 2H).

### FAK Y397E exhibits a higher degree of biological activity than FAK Y397F

**Fig 3:**
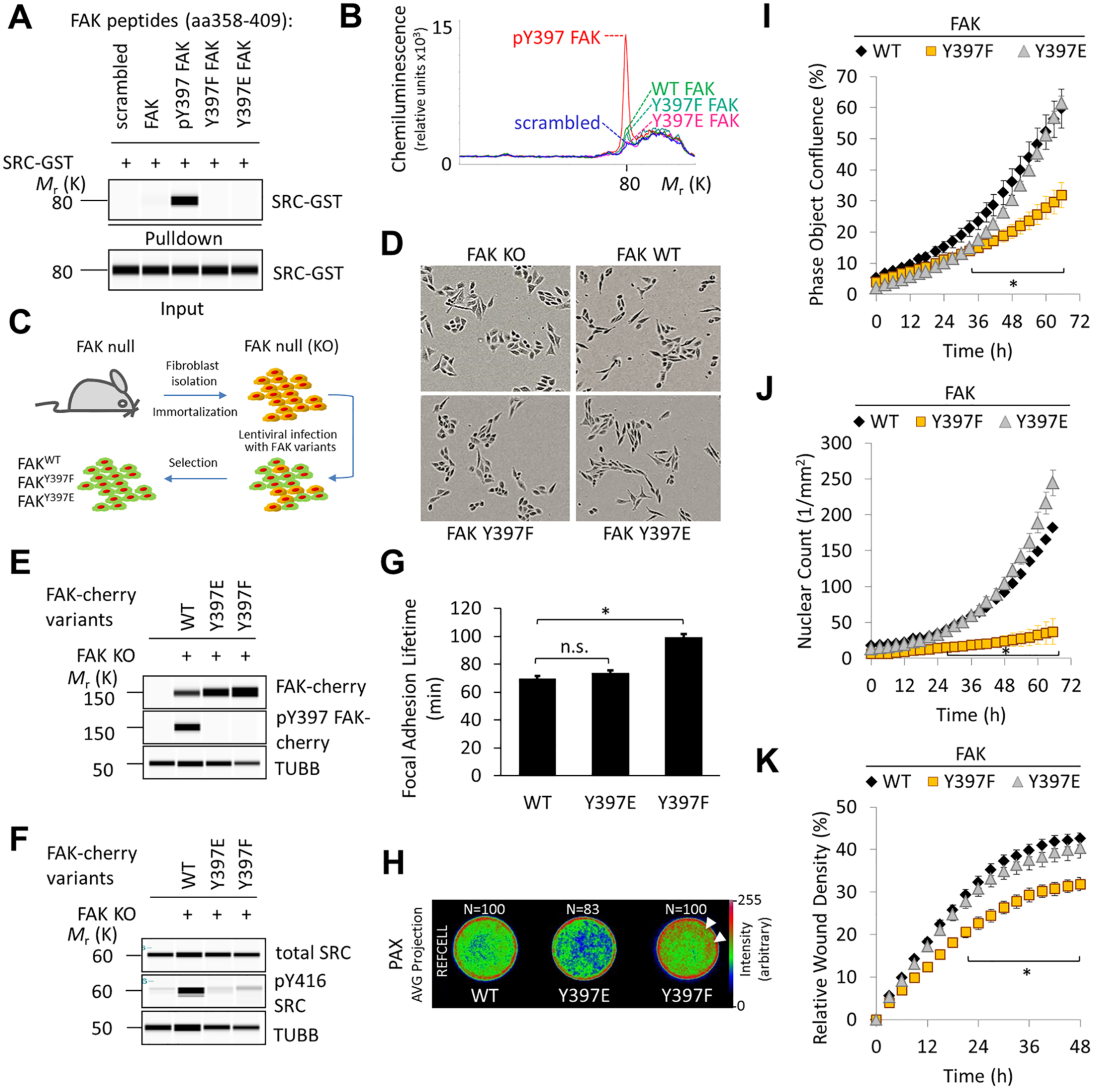
Y397E FAK demonstrates residual activity. (A) Peptide pulldowns using bead-immobilized FERM-kinase linker domain peptides and recombinant SRC-GST. The pulldown blot indicates which samples captured SRC-GST, while the input blot indicates the amount of SRC-GST in each sample prior to experimentation. SRC-GST, SRC tagged with glutathione-s-transferase; M_r_ (K), weight in kilo-Daltons; aa, amino acid. (B) Chemiluminescent signal of SRC-GST bound to peptide in each pulldown sample. (C) FAK-null MEFs were transduced with lentiviral constructs encoding for WT FAK, Y397F FAK, and Y397E FAK. KO, knock-out; WT, wild-type. (D) Phase-contrast images of MEF lines expressing either WT, Y397F, Y397E or no FAK. KO, knock-out. (E) Western blots of FAK, pY397 FAK, and loading control TUBB in lysate of FAK variant MEF lines and FAK-null MEFs. TUBB, tubulin β-chain. (F) Western blots of total SRC, pY416 SRC, and TUBB in lysates of FAK variant MEF lines and FAK-null MEFs. (G) Adhesion turnover measurements of GFP-Paxillin-positive focal adhesions in WT and FAK variant MEFs (mean ± SD, *n* = 180; *P < 0.001, Student’s t test; n.s., not significant). (H) Averaged *N* confocal images of paxillin (PAX)-stained FAK variant cells (reference cells); signal density was processed over the image stack and pseudocolored. Arrows indicate areas of PAX adhesions in low contractility areas. (I) Phase object confluence of FAK variant MEF lines, indicating the percentage of cells covering the field of view over 72 hours. Statistically significant differences between WT FAK and Y397F FAK confluence are indicated below relevant data points (mean ± SEM, *n* = 24; *P < 0.05, Student’s t test; h, hours). (J) Nuclear counts of fluorescently labeled nuclei of FAK variant MEFs are depicted as a measure of cell proliferation (mean ± SEM, *n* = 24; *P < 0.05, Student’s t test). (K) Relative wound density of FAK variant MEF lines indicating the percentage of cells to invade Matrigel following wound treatment. Statistically significant differences between WT and Y397F FAK lines are indicated below relevant data points. Immunoblots are pseudoimages generated by the ProteinSimple Compass software (mean ± SEM, *n* = 12; *P < 0.05, Student’s t test).

Phospho (p)-Y397 FAK has been shown to bind the Src-homologue-2 domain (SH2) of SRC and form a protein complex that is implicated in integrin signaling and cell adhesion [23]. We therefore decided to investigate the binding potential and activity of FAK Y397E to determine whether residual binding with SRC would explain the extended lifespan and advanced development of FAK Y397E versus Y397F embryos. The affinity of recombinant full-length SRC for synthesized peptide corresponding to the FAK linker region, including FAK proline rich region 1 (PRR1) and amino acids (aa) 358–409 harboring modifications at aa 397 was tested in vitro as shown. Peptides also contained FAK proline rich region 1 (aa368-375) which serves as a binding site for SH3 domains including SRC-SH3. Desthiobiotinylated peptide was immobilized to streptavidin-coated beads and incubated with glutathione S-transferase-tagged recombinant SRC (SRC-GST). The amount of precipitated SRC-GST was then determined by microfluidic immunoblotting using the ProteinSimple Wes platform. We found that only the pY397 FAK peptide but not scrambled, nonphosphorylated (FAK), Y397F, or Y397E FAK peptides precipitated SRC-GST (Fig 3A). Quantification of the chemiluminescent signal from pY397 FAK peptide-SRC-GST pulldowns demonstrated a large peak at 80 kDa indicating effective binding of pY397 FAK peptide to SRC-GST. No or minimal signal was seen with scrambled, nonphosphorylated (WT), Y397F or Y397E peptides (Fig 3B) indicating weak or no binding of nonphosphorylated peptides to SRC-GST. These results suggested that efficient FAK binding to full length SRC requires pY397 FAK and cannot be achieved by a negatively charged ‘phospho-mimicking’ glutamate at amino acid 397.

To determine expression and activation of FAK and SRC in culture, we stably transduced FAK-null mouse embryonic fibroblasts (MEFs) with lentiviral constructs encoding for FAK variants (Fig 3C). MEFs expressing FAK WT, FAK Y397F, or FAK Y397E were then seeded on gelatin-coated surfaces where they maintained a classic fibroblast morphology (Fig 3D). Immunoblotting experiments showed that FAK was detected in all three FAK variant lysates. pY397 FAK was however only detected in the WT FAK lysate (Fig 3E) as FAK Y397E did not bind anti-pY397 FAK antibody. SRC was expressed at a similar level in all three FAK variant and knock out (KO) cell lines. In contrast, active SRC as measured by pY416 was abundant in FAK WT lysates, and almost undetectable in KO and FAK Y397F lysates but slightly more abundant in Y397E lysates (Fig 3F). These data suggested that while SRC is present in all tested MEF lines, its activation depends on pY397 FAK.

Since FAK is involved in focal adhesion turnover, wound healing, and survival, assays comparing adhesion, proliferation and motility allowed us to infer the functionality of the variants. FAK variant MEFs were analyzed microscopically for their ability to affect turnover of green fluorescent-paxillin-positive focal adhesions. We found that focal adhesion lifetime was increased almost 2-fold in FAK Y397F MEFs whereas no significant change was detected with FAK Y397E versus WT (Fig 3G). FAK variant fibroblasts were also seeded on fibronectin micropatterned discs and stained for paxillin (PAX), a focal adhesion marker. *N* confocal images of PAX-stained FAK variant cells were averaged and their signal density processed over the image stack and pseudocolored. In all FAK variant cells, PAX-containing adhesions were found in a predominantly circumferential distribution. However, in FAK Y397F cells, PAX-containing adhesions were also found in less contractile areas towards the center of the discs. Such aberrant formation of adhesion structures was thought to impede FAK Y397F cell migration and invasion. We therefore analyzed FAK variant MEFs for their ability to migrate and invade into Matrigel.

Cells were imaged and analyzed for phase object confluence, which measures the percent of the field of view covered by confluent cells. A significant reduction in confluence was seen with FAK Y397F but not Y397E versus WT (Fig 3I). Similarly, primary FAK Y397F MEFs that were directly derived from knock-in mice showed delayed spreading and reduced phase object confluence after plating versus WT (S1 Fig). To more directly measure cell proliferation, FAK variant MEFs were transduced to express a nuclear-restricted red fluorescent protein. This enabled real-time counting of cells. We found that the doubling time of nuclei was reduced in Y397F but not in Y397E MEFs compared to WT (Fig 3J). Likewise, Matrigel invasion following scratch wounding was significantly reduced with FAK Y397F but not Y397E versus WT (Fig 3K). These data showed that FAK Y397E retains a higher degree of biological activity than FAK Y397F. This is further demonstrated by Y397E but not Y397F FAK MEFs maintaining normal osteopontin (SPP1) expression (S2 Fig and S1 Dataset), a downstream read-out of FAK activity [10].

### Skin homeostasis unaltered in conditional Y397F and Y397E mutant mice

**Fig 4:**
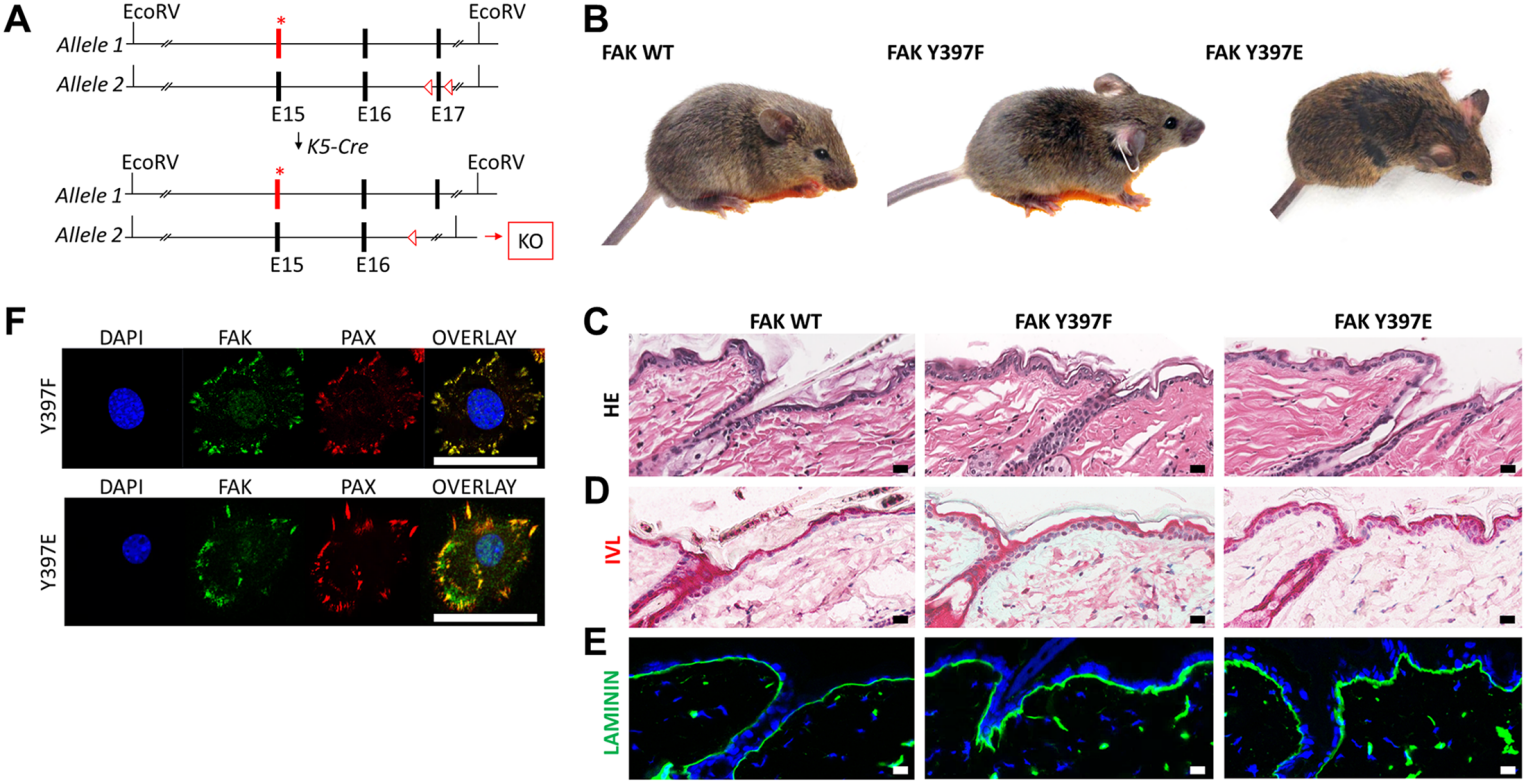
No epidermal phenotype in conditional Y397F FAK and Y397E FAK mice. (A) Heterozygous mice with one FAK allele encoding for Y397F FAK or Y397E FAK and one allele containing floxed exon 17 were crossed with mice expressing Cre recombinase under a K5 promoter to ensure exclusive expression of Y397F FAK or Y397E FAK in the epidermis of offspring. K5, keratin-5. (B) Images of adult WT, Y397F FAK, and Y397E FAK mice. (C) HE staining of WT, Y397F FAK, and Y397E FAK epidermis. HE, hematoxylin and eosin. (D) Immunohistochemical staining of involucrin using an alkaline phosphatase-based red detection kit. (E) Immunofluorescent staining of laminin-332 expression in WT, Y397F FAK, and Y397E FAK epidermis. Laminin shown in green, DAPI shown in blue. DAPI, 4’,6-Diamidine-2’-phenylindole. (F) Immunocytochemical staining of FAK variant epidermal keratinocytes. FAK staining shown in green, paxillin staining shown in red, and DAPI staining shown in blue. PAX, paxillin.

To determine whether Y397F or Y397E FAK would present a phenotype if selectively expressed in the epidermis, FAK mutant mice were crossed with mice expressing Cre recombinase under a keratin 5 promoter (K5-Cre; Fig 4A). Conditional Y397F and Y397E FAK mice retained a normal phenotype. On gross inspection, three week-old mice harboring Y397F or Y397E FAK were indistinguishable from control litter-mates (Fig 4B). No hair phenotype was encountered throughout the entire life span of FAK Y397F or Y397E mice indicating normal hair follicle stem cell function. Histology revealed normal interfollicular epidermis without sub-epidermal blistering or inflammatory infiltrates (Fig 4C). Immunolabeling of laminin 332, a basement membrane marker, and involucrin, a marker of epidermal differentiation, also demonstrated no obvious difference in epidermal or basement membrane architecture (Fig 4D and 4E). FAK mutant protein was expressed in keratinocytes and co-localized with paxillin to focal adhesions (Fig 4F). The unaltered epidermal morphology and expected expression and cellular localization of FAK Y397F and Y397E suggested that Y397 is not necessary for normal epidermal function. This finding is in line with previously reported research, wherein FAK was deleted in keratin-14-expressing basal keratinocytes, resulting in no deleterious phenotype but rather tumor suppression [15].

## Discussion

In the present study, we show that phosphorylated Y397 FAK is necessary for normal embryonic development but dispensable for unchallenged skin homeostasis. Our data suggests that inactivating mutations in Y397 FAK result in decreased expression of FN-like ECM and defective mesenchymal angiogenesis—a process that depends on FN-like ECM [22]–but have no effect on normal epidermis and epidermal appendages which are avascular tissues.

Substituting Y397 with a glutamate or phenylalanine residue in the germline allowed us to investigate the role of Y397 FAK phosphorylation in vivo. Glutamate introduces a negative charge, thus retaining FAK function compared to Y397F, including enhanced embryonic development and reduced alteration of gene expression. However, glutamate is not truly “phospho-mimicking” as it does not fit the binding pocket of SH2 adaptor domains [24]. Specifically, we have shown that SRC SH2 does not interact with Y397E FAK linker peptide. The combination of a constitutive negative charge and a disrupted SH2 binding pocket ultimately results in hemorrhage and embryonic death by E16.5. Interestingly, mice with a deletion of the 19 amino acid FERM-kinase domain linker (Δ15 FAK) which includes Y397 show a phenotype that is similar to Y397E embryos [13]. A shortened linker would likely impair FERM-kinase domain interaction due to structural constraints [25]. Indeed, basal FAK activity is increased by Δ15 deletion [13]. Structural predictions lead to the assumption that the same may be true for FAK Y397E [25].

Y397 FAK plays an important role in complex mesenchymal processes such as angiogenesis and drives the expression of FN-type ECM. FN secretion and matrix assembly in the ECM are necessary for angiogenesis and vasculogenesis, and FN is primarily expressed during development, wound-healing, and tumor formation [26]. FN-integrin binding activates FAK to form focal adhesions and initiate signals to assist in FN-based matrix assembly [27]. Y397F FAK embryos resemble FN-deficient embryos [10], and FAK-null embryos exhibit deficient FN matrices and patterning [28]. These findings help explain why Y397E and Y397F FAK embryos experienced stalled development and impaired angiogenesis, namely that the activity of Y397E and Y397F FAK was insufficient to maintain their roles in FN-based matrix assembly prior to angiogenesis. However, adult skin only expresses FN in the basement membrane [29], and FN-receptors like α5β1 integrin are not expressed at meaningful levels in the epidermis [30]. Therefore, the lack of a phenotype in unchallenged adult skin of Y397E and Y397F FAK mice is expected.

FAK has been shown to promote an immunosuppressive microenvironment that allows cancerous cells to evade immune surveillance [7]. Conditional FAK knock-out reduces the risk of carcinogenesis in breast and skin tissue [14, 15]. FAK Y397 drives FN-type gene expression [10], which in turn protects the stem cell niche [31]. Cancer stem cells overexpress FAK, where it induces stem cell self-renewal and metastasis [32]. FAK likely promotes ECM remodeling and contributes to the pathogenesis of cancer and other prevalent diseases. Future research must provide additional detail on the role of FAK in subcellular compartments that sustain the FAK-induced ECM remodeling response such as the nucleus.

## Supporting information

S1 Fig Reduced cell spreading in primary FAK Y397F MEFs. (A) Wild-type and FAK Y397F mutant MEFs were plated on plastic and monitored by live cell imaging over time. Mutant MEFs were delayed in their ability to spread after attachment. (B-C) Automated quantification of phase object confluence (B) and average phase object area (C) based on a pre-defined confluence mask. Both outcome measures confirm the delayed spreading and phase confluence as observed on images (mean ± SD, n = 24; *P < 0.001, Student’s t test; n.s., not significant; h, hours).
(TIF)

S2 Fig Y397E FAK MEFs exhibit gene expression closer to WT MEFs. (A) Logarithmic change in gene expression of KO, Y397F FAK, and Y397E FAK embryos normalized to wild-type expression. KO/WT expression shown in blue, Y397F FAK/WT expression shown in red, and Y397E/WT expression shown in green. KO, knock-out; WT, wild-type. (B) Expression of FN-associated genes in KO, Y397F FAK, and Y397E FAK embryos normalized to WT expression, with blue indicating decreased expression and red indicating increased expression. FAK Y397F but not Y397E embryos resembled that of fibronectin (FN)-deficient embryos10,18. We therefore hypothesized that FAK Y397F may impact FN-type gene expression to a greater extent than FAK Y397E. FC, fold change.
(TIF)

S1 Dataset List of genes induced by FAK variants as determined by total RNA sequencing. (XLSX)
