# Supplementary figures and images for: FAK auto-phosphorylation site tyrosine 397 is required for development but dispensable for normal skin homeostasis
Source: PLoS One. 2018 Jul 12;13(7):e0200558. doi: 10.1371/journal.pone.0200558 (PMC6042779; doi:10.1371/journal.pone.0200558)

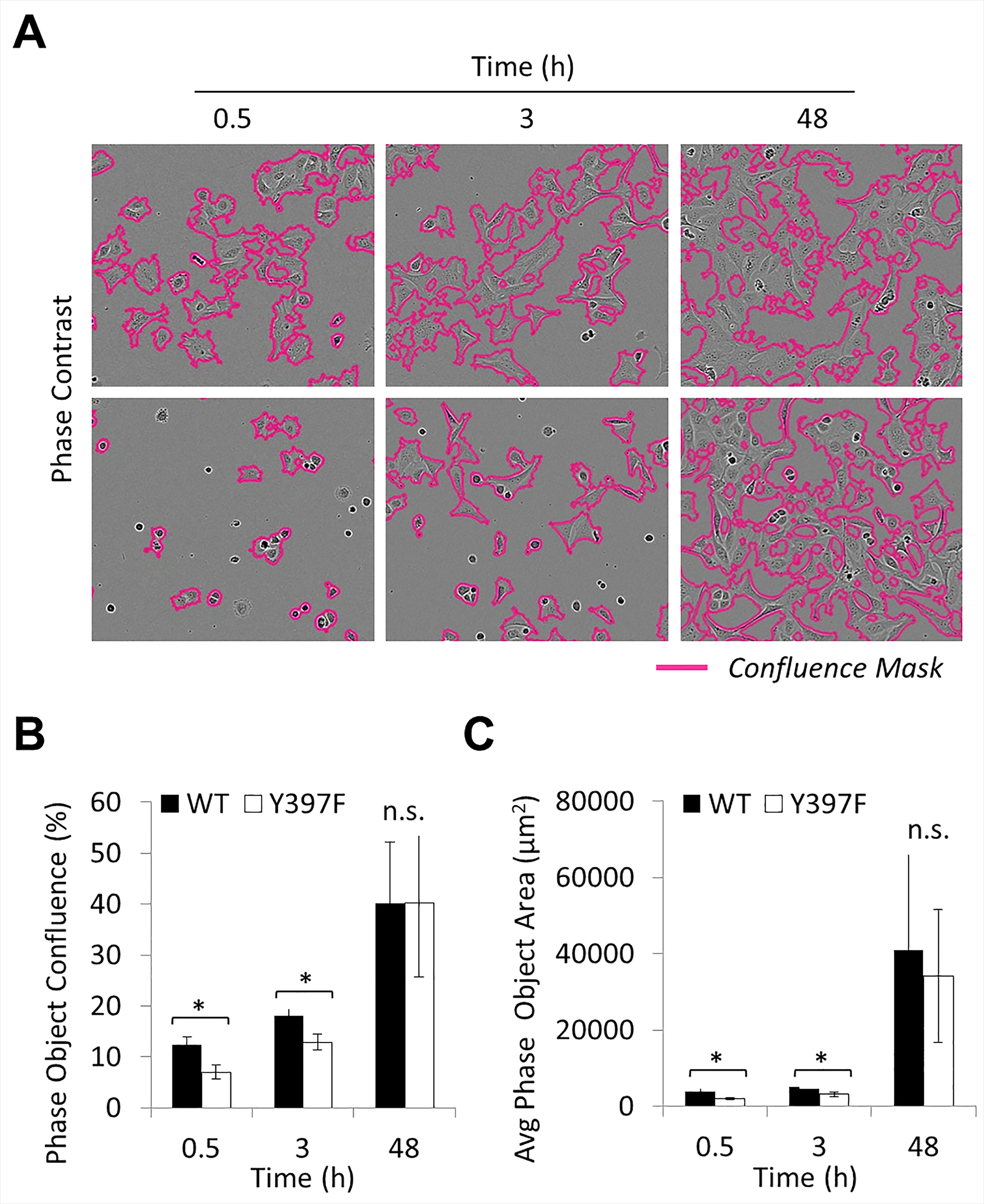

Supplement: S1 Fig — (A) Wild-type and FAK Y397F mutant MEFs were plated on plastic and monitored by live cell imaging over time. Mutant MEFs were delayed in their ability to spread after attachment. (B-C) Automated quantification of phase object confluence (B) and average phase object area (C) based on a pre-defined confluence mask. Both outcome measures confirm the delayed spreading and phase confluence as observed on images (mean ± SD, n = 24; *P < 0.001, Student’s t test; n.s., not significant; h, hours). (TIF) [file pone.0200558.s001.tif]

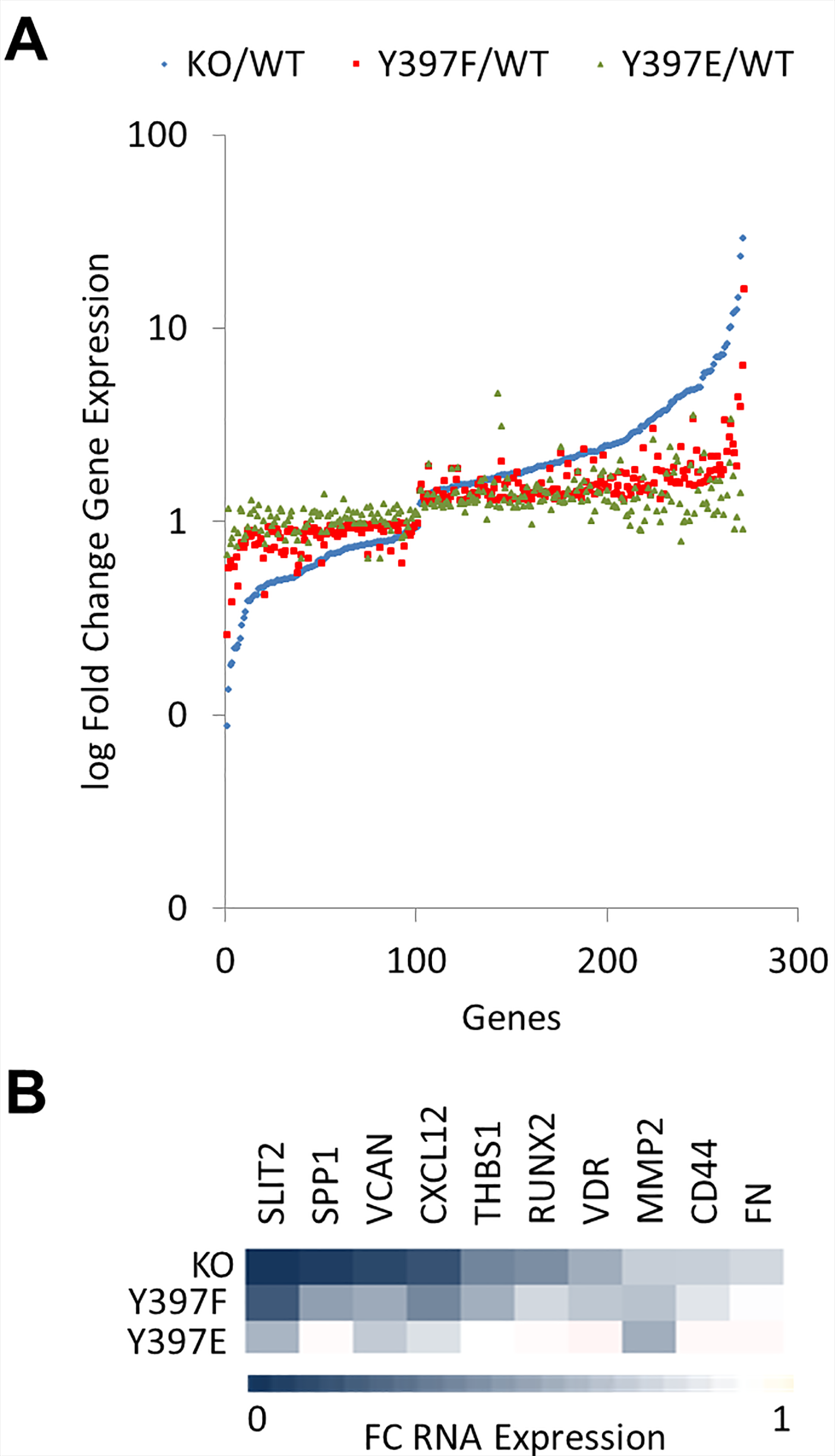

Supplement: S2 Fig — (A) Logarithmic change in gene expression of KO, Y397F FAK, and Y397E FAK embryos normalized to wild-type expression. KO/WT expression shown in blue, Y397F FAK/WT expression shown in red, and Y397E/WT expression shown in green. KO, knock-out; WT, wild-type. (B) Expression of FN-associated genes in KO, Y397F FAK, and Y397E FAK embryos normalized to WT expression, with blue indicating decreased expression and red indicating increased expression. FAK Y397F but not Y397E embryos resembled that of fibronectin (FN)-deficient embryos10,18. We therefore hypothesized that FAK Y397F may impact FN-type gene expression to a greater extent than FAK Y397E. FC, fold change. (TIF) [file pone.0200558.s002.tif]
